# Adolescent gender dysphoria management: position paper from the Italian Academy of Pediatrics, the Italian Society of Pediatrics, the Italian Society for Pediatric Endocrinology and Diabetes, the Italian Society of Adolescent Medicine and the Italian Society of Child and Adolescent Neuropsychiatry

**DOI:** 10.1186/s13052-024-01644-7

**Published:** 2024-04-18

**Authors:** Valeria Calcaterra, Gianluca Tornese, Gianvincenzo Zuccotti, Annamaria Staiano, Valentino Cherubini, Rossella Gaudino, Elisa Maria Fazzi, Egidio Barbi, Francesco Chiarelli, Giovanni Corsello, Susanna Maria Roberta Esposito, Pietro Ferrara, Lorenzo Iughetti, Nicola Laforgia, Mohamad Maghnie, Gianluigi Marseglia, Giorgio Perilongo, Massimo Pettoello-Mantovani, Martino Ruggieri, Giovanna Russo, Mariacarolina Salerno, Pasquale Striano, Giuliana Valerio, Malgorzata Wasniewska, Italian Academy of Pediatrics, Italian Society of Pediatrics, Italian Society for Pediatric Endocrinology and Diabetes, Italian Society of Adolescent Medicine, Italian Society of Child and Adolescent Neuropsychiatry, Massimo Agosti, Carlo Virginio Agostoni, Alessandro Aiuti, Chiara Azzari, Raffaele Badolato, Adriana Balduzzi, Eugenio Baraldi, Roberto Berni Canani, Alessandra Biffi, Andrea Biondi, Gianni Bisogno, Nicola Brunetti Pierri, Virginio Carnielli, Stefano Cianfarani, Paola Cogo, Luigi Corvaglia, Carlo Dani, Giovanni Di Salvo, Franca Fagioli, Vassilios Fanos, Giovanni Battista Ferrero, Ruggiero Francavilla, Luisa Galli, Diego Gazzolo, Carlo Giaquinto, Paola Giordano, Eloisa Gitto, Salvatore Grosso, Alfredo Guarino, Flavia Indrio, Marcello Lanari, Paolo Lionetti, Franco Locatelli, Fortunato Lombardo, Claudio Maffeis, Bruno Marino, Fabio Midulla, Emanuele Miraglia Del Giudice, Michele Miraglia Del Giudice, Giovanni Montini, Giancarlo Parenti, Pasquale Parisi, Diego Peroni, Silverio Perrotta, Giorgio Piacentini, Angelo Pietrobelli, Francesco Raimondi, Ugo Ramenghi, Angelo Ravelli, Claudio Romano, Francesca Rossi, Paolo Rossi, Vincenzo Salpietro Damiano, Alberto Spalice, Agnese Suppiej, Riccardo Troncone, Alberto Verrotti

**Affiliations:** 1 Pediatric Department, Buzzi Children’s Hospital, Milano, Italy; 2 Department of Internal Medicine and Therapeutics, University of Pavia, Pavia, Italy; 3 Department of Medicine, Surgery and Health Sciences, University of Trieste, Trieste, Italy; 4 Department of Pediatrics, Institute for Maternal and Child Health, IRCCS Burlo Garofolo, Trieste, Italy; 5 Department of Biomedical and Clinical Sciences, University of Milan, Via GB Grassi, n.74, Milano, 20157 Italy; 6 Department of Translational Medical Sciences, Section of Pediatrics, University Federico II of Naples, Naples, Italy; 7 Department of Women’s and Children’s Health, Azienda Ospedaliero-Universitaria delle Marche, Ospedali Riuniti di Ancona, “G. Salesi Hospital”, Ancona, Italy; 8 Pediatric Unit, Department of Surgical Sciences, Dentistry, Gynecology and Pediatrics, University of Verona, Verona, Italy; 9 Unit of Child Neurology and Psychiatry, ASST Spedali Civili of Brescia, Brescia, Italy; 10 Department of Clinical and Experimental Sciences, University of Brescia, Brescia, Italy; 11 Department of Pediatrics, University of Chieti-Pescara, Chieti, Italy; 12 Division of Pediatrics, “A.R.N.A.S.” Civic Hospital, Di Cristina Benfratelli, University of Palermo, Palermo, Italy; 13 Pediatric Clinic, Pietro Barilla Children’s Hospital, Department of Medicine and Surgery, University Hospital of Parma, Parma, Italy; 14 Department of Medicine and Surgery, Università Campus Bio-Medico, Roma, Italy; 15 Operative Research Unit of Pediatrics, Fondazione Policlinico Universitario Campus Bio-Medico, Roma, Italy; 16 Paediatric Unit, Department of Medical and Surgical Sciences of Mothers, Children and Adults, University of Modena and Reggio Emilia, Modena, Italy; 17 Section of Neonatology and Neonatal Intensive Care Unit, Interdisciplinary Department of Medicine (DIM), University of Bari “Aldo Moro”, Bari, Italy; 18 IRCCS Istituto Giannina Gaslini, Genoa, Italy; 19 Department of Neuroscience, Rehabilitation, Ophtalmology, Genetics, Maternal and Child Health, University of Genoa, Genoa, Italy; 20 Department of Clinical, Surgical, Diagnostic and Pediatric Sciences, Pediatric Unit, University of Pavia, Pavia, Italy; 21 Pediatric Clinic, Fondazione IRCCS Policlinico San Matteo, Pavia, Italy; 22 Department of Rare Diseases, University Hospital of Padua, Padua, Italy; 23 Division of Pediatrics, Department of Woman’s & Child’s Health, University Hospital of Padua, Padua, Italy; 24 Institute for Scientific Research «Casa Sollievo», University of Foggia, Foggia, Italy; 25 Unit of Pediatric Clinic, Centre for Rare Diseases of the Nervous System in Childhood, Department of Clinical and Experimental Medicine, University of Catania, Catania, Italy; 26 Pediatric Hematology and Oncology Unit, Department of Clinical and Experimental Medicine, University of Catania, Catania, Italy; 27 Department of Translational Medical Sciences, Paediatric Endocrinology Unit, University “Federico II”, Naples, Italy; 28 Department of Medical, Movement and Wellbeing studies, University of Napoli “Parthenope”, Napoli, Italy; 29 Department of Human Pathology in Adulthood and Childhood, University of Messina, Messina, Italy

**Keywords:** Gender dysphoria, GnRH analogs, Gender incongruence, Gender-affirming hormone therapy, Gender-affirming care, Pediatrics, Adolescence

## Abstract

**Background:**

In response to the imperative need for standardized support for adolescent Gender Dysphoria (GD), the Italian Academy of Pediatrics, in collaboration with the Italian Society of Pediatrics, the Italian Society for Pediatric Endocrinology and Diabetes, Italian Society of Adolescent Medicine and Italian Society of Child and Adolescent Neuropsychiatry is drafting a position paper. The purpose of this paper is to convey the author's opinion on the topic, offering foundational information on potential aspects of gender-affirming care and emphasizing the care and protection of children and adolescents with GD.

**Main body:**

Recognizing that adolescents may choose interventions based on their unique needs and goals and understanding that every individual within this group has a distinct trajectory, it is crucial to ensure that each one is welcomed and supported. The approach to managing individuals with GD is a multi-stage process involving a multidisciplinary team throughout all phases. Decisions regarding treatment should be reached collaboratively by healthcare professionals and the family, while considering the unique needs and circumstances of the individual and be guided by scientific evidence rather than biases or ideologies. Politicians and high court judges should address discrimination based on gender identity in legislation and support service development that aligns with the needs of young people. It is essential to establish accredited multidisciplinary centers equipped with the requisite skills and experience to effectively manage adolescents with GD, thereby ensuring the delivery of high-quality care.

**Conclusion:**

Maintaining an evidence-based approach is essential to safeguard the well-being of transgender and gender diverse adolescents.

## Background

"Sex," or "natal gender," refers to a label, commonly "male" or "female," that is usually assigned at birth based on genetic and anatomical characteristics, including genital anatomy, chromosomes, and sex hormone levels. Conversely, "gender identity" is an individual's internal perception of their own identity, arising from a complex interplay of biological traits, developmental influences, and environmental factors. This identity may align with being male or female, fall somewhere in between, represent a combination of both, or exist outside the binary framework of gender altogether.

Gender identity, a fundamental aspect of self-perception, begins to emerge in early childhood and evolves throughout adolescence [1, 2]. Recent research indicates a growing number of transgender and gender diverse (TGD) young individuals identifying with a gender different from their assigned one at birth [3–6] Consequently, there's an escalating demand for gender-related care within pediatric healthcare systems. This demographic increasingly seeks assistance from pediatricians, pediatric endocrinologists, and primary care providers for gender-related and general health needs.

Gender-affirming care, which encompasses psychological, social, medical, and/or surgical interventions, necessitates a multidisciplinary approach at every stage [7].

Hormonal treatment may play a vital role in comprehensive gender-affirming care for some TGD individuals, sparking extensive debate and ethical discussions, particularly regarding its use in adolescents under 18. While concerns about the lack of evidence for such treatments persist, recent clinical evidence supports medical intervention in gender dysphoria (GD), challenging the neutrality of omitting or delaying treatment.

However, potential effects of suppressive treatment with GnRH analogs (GnRHa) on various aspects such as growth, skeletal development, neurodevelopment, fertility, and future surgical outcomes must be considered [8–17], as with any medical intervention. The National Board of Health and Welfare in Sweden recently introduced updated guidelines for health services catering to children and adolescents with GD. The board stated that, as a collective, the risks associated with puberty blockers and gender-affirming treatment for adolescents with GD are likely to outweigh the anticipated benefits. They emphasized that decisions regarding treatment should be made on a case-by-case basis for each individual [18]. Therefore, facilitating fully informed discussions with TGD youth and families about the risks, benefits, and uncertainties of treatment options is crucial [19].

In response to the need for standardized support, the Italian Academy of Pediatrics (IAP), in collaboration with the Italian Society of Pediatric (ISP), the Italian Society for Pediatric Endocrinology and Diabetes (ISPED), Italian Society of Adolescent Medicine (ISAM) and Italian Society of Child and Adolescent Neuropsychiatry (ISCAN) is drafting a position paper. This paper aims to provide foundational information on potential components of gender-affirming care and ensure the care and protection of children and adolescents with GD, recognizing that individual adolescents may or may not choose these interventions based on their unique needs and goals.

## Main text

### Terminology

Initiating the discussion on sexual identity, a glossary serves as a foundational tool by offering definitions and elucidations to enrich the understanding and application of key terms. These terms encompass biological gender, gender identity, gender role, and sexual orientation, each outlined for significance in Table 1.

**Table 1:** Key terms related to gender, gender identity, expression, and sexuality [20]

**Term**	**Significance**
**Sex assigned at birth**	Refers to a person’s status as male, female, or intersex based on physical characteristics. Sex is usually assigned at birth based on appearance of the external genitalia. AFAB is an abbreviation for “assigned female at birth.” AMAB is an abbreviation for “assigned male at birth.”
**Gender**	Depending on the context, gender may reference gender identity, gender expression, and/or social gender role, including understandings and expectations culturally tied to people who were assigned male or female at birth.
**Gender identity**	Refers to a person’s deeply felt, internal, intrinsic sense of their own gender. Gender identities other than those of men and women (who can be either cisgender or transgender) include transgender, nonbinary, genderqueer, gender neutral, agender, gender fluid, and “third” gender, among others; many other genders are recognized around the world.
**Gender expression**	Refers to how a person enacts or expresses their gender in everyday life and within the context of their culture and society. Expression of gender through physical appearance may include dress, hairstyle, accessories, cosmetics, hormonal and surgical interventions as well as mannerisms, speech, behavioral patterns, and names. A person’s gender expression may or may not conform to a person’s gender identity.
**Sexual Orientation**	Refers to one of different dimensions of the person’s sexual identity, and refers to attractions, and behaviors in relation to people on the basis of their gender(s) and or sex characteristics and those of their partners. Sexual orientation and gender identity are distinct terms.
**Cisgender**	Refers to people whose current gender identity corresponds to the sex they were assigned at birth.
**Transgender* (or trans)***	Umbrella terms used to describe people whose gender identities and/or gender expressions are not what is typically expected for the sex to which they were assigned at birth. These words should always be used as adjectives (as in “trans people”) and never as nouns (as in “transgenders”) and never as verbs (as in “transgendered”).
**Gender diverse**	Term used to describe people with gender identities and/or expressions that are different from social and cultural expectations attributed to their sex assigned at birth. This may include, among many other culturally diverse identities, people who identify as nonbinary, gender expansive, gender nonconforming, and others who do not identify as cisgender.

Gender identity and sexual orientation, often conflated, represent distinct constructs that elude both professionals and laypersons alike. These two dimensions are, along with gender expression and sex assigned at birth part of the multidimensional construct of sexual identity [20]. Gender identity refers to an individual's internal perception of being a boy/man, a girl/woman, a blend of both, or neither, whereas sexual orientation concerns one's emotional and sexual attraction to others. Typically, gender identity begins to manifest earlier in life, often during toddlerhood, whereas awareness of sexual orientation typically emerges during early adolescence [21, 22].

According to Kohlberg’s theory of gender development, the process of self-identification as either a girl or boy unfolds across three stages [23]. At approximately 2–3 years of age, children grasp the concept of labeling themselves and others by sex (gender labeling). By ages three to four, they comprehend the stability of gender over time (gender stability). Finally, around ages four to five, children acknowledge that their sex remains constant across various situations (gender persistence) [24]. As children's sense of gender identity matures, they often exhibit increased attention to individuals of the same sex and aspire to emulate members of their own gender. However, a minority of children may develop a gender identity discordant with their assigned sex at birth.

Subsequently, an alternative theory emerged, proposing that gender stereotypes arise as a method of simplifying the vast amount of information encountered. Essentially, in the intricate world perceived by children, categorizing based on gender serves as a convenient strategy. Initially, children tend to rigidly adhere to these stereotypes, but as they mature, they become progressively more flexible [25]. Furthermore, according to Bandura's theory, a child's gender development is shaped by intricate interactions among behaviors, personal attributes, and environmental influences [26].

### Definitions

When children and adolescents exhibit gender identity or expression incongruent with their biological sex, several main categories are recognized:

- Childhood Gender Diversity (CGD): Defined in the Standards of Care Version 8 by the World Professional Association for Transgender Health (WPATH) [20] CGD describes a phenomenon where prepubescent children display behaviors or expressions not typical for their assigned biological sex. It encompasses children who exhibit cross-gender behaviors or behaviors deviating from the expected sociocultural norms of their gender [27]. CGD is regarded as a natural variation of human gender expression.
- Gender Incongruence (GI): This term, used in the International Classification of Diseases-11th Revision (ICD-11), denotes a marked incongruence between an individual's experienced or expressed gender and their assigned sex [28]. The diagnosis of GI is not solely based on gender-variant behavior and preferences. Since 2018, GI has been reclassified out of mental health categories and into the section "Persons encountering health services in other circumstances" to reduce stigma associated with the condition and to grant the TGD persons the access to health pathways to affirm themselves, if needed.

- GI of Childhood: In prepubertal children (Tanner Stage 1), GI entails:
  1. A strong desire to be a different gender than the assigned sex.
  2. A strong dislike of the child’s own sexual anatomy or anticipated secondary sex characteristics, and/or a strong desire for primary and/or anticipated secondary sex characteristics that align with the experienced gender.
  3. Engagement in make-believe or fantasy play, toys, games, or activities, and preference for playmates typical of the experienced gender rather than the assigned sex. The incongruence must persist for about two years and cannot be diagnosed before the age of five.

- GI of Adolescence (and Adulthood): Characterized by marked and persistent incongruence between an individual's experienced gender and assigned sex, this often leads to a desire to "transition" - to live and be acknowledged as a person of the experienced gender. This transition may involve hormonal treatment, surgery, or other healthcare services to align the individual's body with their desired gender identity and to the extent possible, with the experienced gender. The diagnosis cannot be assigned before the onset of puberty.

- Gender dysphoria (GD) is formally diagnosed in the Diagnostic and Statistical Manual of Mental Disorders-5th Edition (DSM-5) and is characterized by a marked incongruence between one's experienced or expressed gender and their assigned gender, persisting for a minimum of six months. Additionally, to meet the diagnostic criteria, this incongruence must be accompanied by clinically significant distress or impairment in social, occupational, or other important areas of functioning [29]. The criteria for diagnosis differ between children and adolescents/adults.

- In children, GD is identified by the manifestation of at least six of the following criteria (with the first criterion being mandatory):
  1. A strong desire to be of the other gender or insistence on being another gender (or a gender different from one's assigned gender).
  2. In boys (assigned gender), a strong preference for cross-dressing or mimicking female attire; or in girls (assigned gender), a strong preference for exclusively wearing typical masculine clothing and significant resistance to typical feminine attire.
  3. A strong inclination towards cross-gender roles in make-believe or fantasy play.
  4. A strong preference for toys, games, or activities stereotypically associated with the opposite gender.
  5. A strong preference for playmates of the opposite gender.
  6. In boys (assigned gender), a strong rejection of typically masculine toys, games, and activities, coupled with a notable avoidance of rough-and-tumble play; or in girls (assigned gender), a strong rejection of typically feminine toys, games, and activities.
  7. A significant dislike of one's sexual anatomy.
  8. A strong desire for physical sex characteristics that align with one's experienced gender.

- In adolescents (and adults), GD is identified by the presence of at least two of the following criteria:
  1. A marked incongruence between one's experienced or expressed gender and primary and/or secondary sex characteristics (or, in young adolescents, anticipated secondary sex characteristics).
  2. A strong desire to remove one's primary and/or secondary sex characteristics due to a marked incongruence with one's experienced or expressed gender (or, in young adolescents, a desire to prevent the development of anticipated secondary sex characteristics).
  3. A strong desire for primary and/or secondary sex characteristics of the opposite gender.
  4. A strong desire to be of the other gender (or some alternative gender different from one’s assigned gender)
  5. A strong desire to be treated as the other gender (or some alternative gender different from one’s assigned gender)
  6. A strong conviction that one has the typical feelings and reactions of the other gender (or some alternative gender different from one’s assigned gender)

It should be clarified whether the aforementioned criteria also encompass individuals with disorders of sex development (e.g., congenital adrenal hyperplasia or androgen insensitivity syndrome).

According to the DSM-5, it is explicitly stated that 'gender non-conformity is not inherently a mental disorder.' Therefore, gender variance itself is not pathological, but rather dysphoria arises from the distress caused by incongruence between one's body and mind and/or the societal marginalization of gender-variant individuals [27]. Being transgender or gender variant implies no impairment in judgment, stability, reliability, or general social or vocational capabilities; however, these individuals often experience discrimination due to a lack of civil rights protections for their gender identity or expression. Such discrimination damages to the mental health of transgender and gender variant individuals.

Healthcare providers and others who work with and advocate for youth are encouraged to acknowledge the unique needs of this population and serve as allies by striving to establish safe spaces for young people. Furthermore, families, school personnel, and community members can offer essential support to mitigate challenges, to promote the healthy development and to protect the human rights of these youth [30].

### Population estimates

The proportion of TGD children and adolescents is reported to range from 1.2% to 2.7%, depending on factors such as the composition of the study cohort, age demographics, and methodological approach [20]; including broader manifestations of gender diversity, such as gender incongruence or gender ambivalence, the corresponding proportions are higher (8.4% among children and adolescents) [20].

In recent years, there has been a notable increase in the number of children and adolescents seeking support for GD and GI [3–6]. However, this increase is likely only apparent and presumably a result of a decrease in social stigma, which is associated with an improvement in knowledge and services [31]. Notably, not all individuals with GI characteristics in childhood necessarily exhibit symptoms of GD. A notable shift in the sex ratio of clinically referred adolescents has accompanied the increase in referrals, with more assigned female at birth (AFAB) young people now seeking help compared to assigned male at birth (AMAB) [5].

GD can have an early onset and manifest as early as preschool age with variable clinical outcomes [32]. Studies suggest that the majority of gender diverse children (up to 84%) revert to the gender congruent with the sex assigned at birth when they reach puberty (so called *“desisters”*) [33].

Conversely, in the case of adolescents where GD is persistent since childhood and worsens with the onset of puberty (*“persisters”)*, it is less likely to resolve without any gender affirmation intervention. It should be emphasized, however, that in various adolescents with GD, there are no previous instances of non-conforming gender behaviors in childhood [34].

### Rationale for medical treatment in adolescents

GD in adolescence is often associated with emotional and behavioral issues, an increased risk of substance abuse [35–40], self-harm [38], a high suicide rate [38, 41], and the co-occurrence of psychiatric problems (depression, anxiety, dissatisfaction with body image, low self-esteem, dissociative symptoms) [42], autism spectrum disorders [43–45], as well as dissatisfaction in personal and social relationships [46]. Violence and victimization, including sexual assault, dating violence, and bullying, are also common experiences for young individuals with GD [47, 48].

According to reports, the onset of puberty, with its significant bodily changes, marks the most pivotal and distressing period for individuals grappling with GD [49]. Adolescence itself represents a particularly sensitive phase in the journey of self-identification and identity formation [50]. For adolescents with GD, this period presents even more intricate challenges as they navigate the transition from childhood to adulthood while coping with the incongruence between their gender identity and their changing bodies. Puberty is often experienced by adolescents with GD as a natural disaster, disrupting their bodily and gender integrity [49]. The physical transformations during puberty create hurdles for adolescents in maintaining alignment with their experienced gender identity.

A cautious "wait and see" approach with regard to the medical treatment during pre-pubertal years is recommended by guidelines, as in many cases of early-onset GD, the condition tends to regress with the onset of puberty [33]. Families should receive support in comprehending the potential transient nature of this condition, challenging their personal stigmas surrounding gender variance, and emphasizing the significance of fostering a supportive parent-child relationship. Medical treatment is guided by the assessment of goals and the evaluation of risks and benefits. Pubertal adolescents may experience relief through the reduction of secondary sex characteristics such as breasts and facial hair. Currently, there is a lack of evidence-based guidelines for psychological support for children and adolescents with GD. Psychological interventions aimed at altering gender identity have been found to be ineffective and are widely considered unethical [51]. Although there is a shortage of rigorous studies comparing psychotherapy, cognitive behavioral therapy-based approaches, and psychosocial therapy for anxiety and depression, which are the two most prevalent comorbid diagnoses, they could still be provided as treatment options. Pubertal suppression with GnRHa appears to be an effective means to mitigate distress in these cases.

### Therapeutic approach

The therapeutic approach to managing individuals with GD is a multi-stage process involving a multidisciplinary team throughout all phases. The comprehensive pathway may include gender-related care and social transition, puberty suppression (PS), gender-affirming hormonal treatment (GAHT), and gender-affirming surgery

#### Stage 1: gender-related care and social transition

The management of individuals with GD, benefiting from the expertise of a multidisciplinary team during the initial diagnostic phase, should be comprehensive [20, 52]. As recommended by Italian Medicines Agency (AIFA), this team should comprise various professionals such as child and adolescent neuropsychiatrists, pediatric endocrinologists, developmental psychologists, and bioethicists to determine the most suitable treatment plan [53]; however, such a multi-specialist team is not required by the WPATH [20] or the Endocrine Society [54]. Guidelines advocate against immediate therapy initiation, instead recommending psychological follow-up to monitor progress and provide support to the individual and their family. This approach emphasizes a thorough exploration of gender identity [20, 52].

The multidisciplinary team should convene regularly, and after excluding alternative diagnoses, establish therapeutic goals collaboratively.

The initial stage of the therapeutic process, known as the 'social transition,' involves permitting the adolescents to live in their own affirmed gender, informing individuals within the child/adolescent's social circle about their decision to embark on a gender transition. During this phase, the individual may select a name corresponding to their gender identity and adopts the social role associated with that gender, including clothing and behavior [20, 52, 55].

The timing for commencing this social transition should be agreed upon collaboratively between the healthcare professionals and the family, taking into consideration individual sensitivities and prioritizing the overall psychophysical well-being of the individual [55]. Based on the high desistence rates, some advise being cautious in allowing young children to present in their affirmed gender. The worry is that social transition may make it difficult for children to de-transition and thus increase the odds of later unnecessary medical transition [56]. However, there is no evidence that social transition per se leads to unnecessary medical transition. Thus social transition should be seen as a tool to determine the appropriate trajectory for each individual child, with desistence being one potential outcome [56].

In cases of pubertal onset or persistent GD, pharmacological puberty suppression therapy may be considered [20, 52].

#### Stage 2: puberty suppression

In accordance with guidelines, the use of GnRHa to suppress the reproductive axis is deemed appropriate for halting the development of secondary sexual characteristics incongruent with the individual's gender, thereby alleviating distress associated with GD [20, 52].

The decision to initiate puberty suppression therapy should be carefully deliberated by the multidisciplinary team, with consideration given to the unique needs and circumstances of the individual with GD.

The Standards of Care by WPATH outline specific criteria for administering puberty-blocking agents:

1. Persistent and significant gender diversity/incongruence over time.
2. Meeting diagnostic criteria for gender incongruence where necessary for accessing healthcare.
3. Demonstrating emotional and cognitive maturity for informed consent/assent.
4. Addressing any mental health concerns that may affect diagnostic clarity or consent for treatment.
5. Being informed about reproductive implications, including potential fertility loss and options for preservation.
6. Reaching Tanner stage 2 of pubertal development.

Treatment typically involves administering 3.75 mg of intramuscular triptorelin every 28 days, initiating gonadotropin suppression after an initial flare-up. A supplemental dose may be given in the first month to expedite receptor desensitization.

Therapy generally starts between the ages of 11-15 (mean age 14.5±1 years) and continues until around age 16 when GAHT typically commences (mean age 16.2±1 years) [57]. If adolescents need to affirm themselves in their assigned gender at birth, discontinuing GnRHa treatment will result in the resumption of their biological puberty. Suspension of pharmacological treatment may also occur if individuals and their families do not adhere to the psychological process or miss appointments with endocrinologists.

As detailed in Table 2, monitoring before and during GnRHa therapy typically involves assessments every 3-6 months for the first year. This includes auxological measurements (weight, height, body mass index, pubertal assessment), blood pressure checks, and hormonal evaluations (LH, FSH, estradiol/testosterone, prolactin) alongside assessments of bone health such as bone age, bone density evaluations and body composition through Dual Energy X-rays Absorptiometry (DEXA) scans, to be conducted every 12 months and as needed based on clinical requirements [52, 58].

**Table 2:** Clinical, biochemical and instrumental assessments before and during treatment with GnRH analogue

**Assessments prior and during treatment with GnRHa**				
**Clinical, biochemical and imaging evaluation**	**Before starting GnRHa**	**During treatment with GnRHa**		
		**Every 3-6 months**	**Every 6 months**	**Every 12 months**
Weight, height, height velocity, weight, body mass index	X	X		
Bone age (in those who have not completed puberty)	X			X^a^
Pubertal assessment	X		X	
Blood pressure	X	X		
Haemoglobin/haematocrit, ferritin, liver function, renal function, electrolytes	X			
LH, FSH, estradiol/testosterone	X		X	
Prolactin^a^	X		X	
Vitamin D, PTH^a^, calcium, phosphate, albumin	X		X	
BMD (and VFA) by DXA	X			X

*LH* Luteinizing hormoneD *FSH *Follicle-stimulating hormone, *PTH *Parathyroid Hormone, *BMD* Bone mineral density, *DXA* Dual-energy X-ray absorptiometry *VFA* Vertebral fracture assessment

^a^recommended but not essential

In case of vitamin and microelement deficiency and/or sign of decreased bone mineralization, supplementary therapy should be started.

Before commencing GnRHa therapy, individuals who have reached post-pubertal stages may be offered the option of sperm or oocyte retrieval and preservation. In birth-assigned males at Tanner stage 3, ejaculation or electro-ejaculation may yield adequate sperm for storage. For birth-assigned females, oocyte harvesting is only feasible if they have experienced menarche.

For young individuals who begin GnRHa therapy at Tanner stage 2 and proceed with gender-affirming hormones, neither sperm production nor menstruation will occur, making gamete cryopreservation impractical. If individuals later decide to preserve fertility post-GnRHa initiation, it may take 6 months or more for the reproductive axis to recover, with reproductive capacity similar to that at the onset of treatment. However, many young individuals and families, after informed consent, may opt not to pursue fertility preservation.

#### Stage 3: gender-affirming hormonal treatment

Following GnRHa treatment, synthetic sex steroids are introduced to induce the development of secondary sexual characteristics associated with the affirmed gender. Criteria for accessing GAHT align with those for GnRHa treatment [20]. GAHT might also be started without previous GnRHa treatment in adolescents older than 16 years with completed puberty [59]; in this case, preservation options should also be offered before starting treatment.

Two treatment regimens are considered: induction of a "new" puberty with doses typical for prepubertal hypogonadal adolescents if GnRHa treatment commenced during early Tanner stages, or initiation at higher doses gradually escalated for physically mature individuals. GnRHa treatment need not be administered in supraphysiological doses to suppress endogenous sex steroid production [52].

For transgender girls, natural 17-beta-estradiol is preferred over synthetic estrogens due to its lower thrombogenic risk. The initial dosage is 5 mcg/kg/day, incrementally increased every 6 months until reaching a maintenance dosage of 2–4 mg. In transgender girls who initiated treatment in the late pubertal stage, estrogens can be initiated at a dosage of 1 mg, followed by an increase to 2 mg after 6 months. Transgender girls require continued gonadal suppression until gonadectomy. GnRHa are preferred over other anti-androgens such as cyproterone acetate or spironolactone [52]. As there is no available data on the effectiveness of exogenous synthetic sex steroids in suppressing the gonadal axis during puberty, GnRHa should be continued until gonadectomy, especially when initiated in early puberty

For transgender boys, testosterone esters injections are recommended for pubertal induction. The starting dosage is 25 mg/m^2^ every 2 weeks intramuscularly, increased to 25 mg/m^2^ every 6 months. The maintenance dosages vary from 200 mg per 2 weeks for testosterone monoesters, such as testosterone enanthate, to 250 mg im per 3–4 weeks for testosterone esters mixture [52]. For transgender boys who initiated treatment in the late pubertal stage, testosterone can be initiated at a dosage of 75 mg intramuscularly every 2 weeks, followed by the maintenance dosage after 6 months. It is recommended to maintain GnRHa treatment at least until the maintenance dosage of testosterone is reached and preferable to continue until gonadectomy [52].

During this phase of the transition process (and subsequently during adulthood, as hormone therapy is lifelong), it is necessary to undergo regular monitoring, both from a clinical and laboratory/instrumental standpoint. Although hormone therapy in transgender individuals is safe when conducted under close medical supervision, the onset of side effects cannot be excluded, and patients must be adequately informed before starting it. Among the side effects to consider are decreased insulin sensitivity, weight gain, slight increases in hemoglobin and hematocrit, and decreased HDL cholesterol in transgender males undergoing testosterone therapy [54, 60–62]. It should be noted that an increased risk of thromboembolic events has been reported in male-to-female-transitioning individuals [54, 60–62].Nevertheless, all subjects should be informed about the effects of hormone treatment on fertility, particularly regarding the prolonged exposure of testicles to estrogen, which has been associated with testicular damage, and the uncertain effect of prolonged treatment with exogenous testosterone on ovarian function.

#### Stage 4: gender-affirming surgery

The aim of gender-affirming surgery is to align physical characteristics with the affirmed gender, with genital appearance as natural as possible. However, not all transgender or gender-incongruent individuals seek surgical interventions.

As per guidelines from the WPATH [20], criteria for gender-affirming surgery include:

1. Persistent and significant gender diversity/incongruence over time.
2. Meeting diagnostic criteria for gender incongruence when necessary for healthcare access.
3. Demonstrating emotional and cognitive maturity for informed consent/assent.
4. Addressing any mental health concerns that may affect diagnostic clarity or consent for treatment, ensuring optimal provision of gender-affirming medical treatment.
5. Being informed about reproductive implications, including potential fertility loss and available fertility preservation options.
6. Undergoing at least 12 months of gender-affirming hormone therapy, or longer if needed, to achieve desired surgical outcomes for gender-affirming procedures, such as breast augmentation, orchiectomy, vaginoplasty, hysterectomy, phalloplasty, metoidioplasty, and facial surgery [52].

Generally, gender-affirming surgery is recommended after the age of 18 [20].

Common surgical procedures for trans men include mastectomy, genital surgeries like salpingo-oophorectomy and hysterectomy, and neopenis creation with implantation of erectile and testicular prostheses. For trans women, typical surgeries include breast augmentation, facial feminization procedures, thyroid cartilage reduction, and genital surgeries like gonadectomy, penectomy, and neovagina creation [52].

### Focus on the use of GnRH agonist in adolescents with gender dysphoria

The utilization of suppressive therapy with GnRH remains a highly debated aspect in the treatment of GD in pediatric patients. Hence, it is considered pertinent to dedicate special attention to this topic within the framework of this position paper.

#### Details on the introduction of GnRHa treatment in gender dysphoria

The initial protocol for using GnRHa in adolescents originated in the Netherlands in the 1990s, often referred to as "the Dutch protocol" or "the Dutch approach." It combines psychological support with medical interventions [59, 63]. Since then, pubertal suppression (PS) with GnRHa has been recommended by major national and international scientific societies [20, 54]. The first Italian paper on PS in adolescents with gender dysphoria, endorsed by national scientific societies, was published a decade ago [64].

During the initial phase of seeking medical assistance, individuals undergo psycho-diagnostic assessment for gender dysphoria. Eligible adolescents undergo PS as part of an extended diagnostic process [64]. GnRHa act by temporarily desensitizing the GnRH receptor, allowing for spontaneous resumption of the hypothalamic-pituitary-gonadal axis after discontinuation [41, 65]. This treatment temporarily halts further pubertal progression, providing time for diagnostics and mental health evaluation, with extensive experience in young children with central precocious puberty [65], where pubertal suppression is fully reversible.

The potential benefits of using GnRHa, as outlined by the Italian Committee for Bioethics [66] in 2018, include:

1. Enabling the medical team to "widen the diagnostic window," allowing for a more comprehensive exploration of issues related to the adolescent's gender identity and facilitating the maturation of the individual's awareness, free from the discomfort associated with pubertal development.
2. Prevention of irreversible physical changes of puberty in those in early puberty (Tanner stage 2-3), which can cause significant distress for adolescents with gender dysphoria.
3. Providing the opportunity, if the adolescent proceeds to affirming medical interventions, to avoid physical changes, potentially reducing the need for hormones and less invasive surgical procedures in the future (>16 years) and preventing adolescents from resorting to risky behaviors such as self-administration of online-purchased drugs without proper oversight and monitoring.

Furthermore, since GnRHa do not induce permanent physical changes, they serve as a reversible intervention if the adolescent decides to detransition or retransition, allowing for the restoration of sexual development according to the sex assigned at birth.

GnRHa can be also initiated in Tanner stage 4–5 of puberty or in post-pubertal adolescents, halting menstruation or erections and potentially preventing further development of secondary sex characteristics. As a matter of fact, the mean age at start of GnRHa is quite late (14.0 years for AMAB and 15.5 for AFAB adolescents), and only 4.6% of AFAB adolescents start GnRHa in early puberty compared to 34% of AMAB [67]. However, GnRHa treatment is not recommended for prepubescent children.

The effectiveness of GnRHa treatment has been demonstrated in studies such as the longitudinal study on the first 70 adolescents who underwent puberty suppression in Amsterdam between 2000 and 2008. This study showed improvements in psychological functioning and a reduction in behavioral and emotional problems and depressive symptoms during puberty suppression [68]. Subsequent research on individuals from the same cohort, assessed one year after gender-reassignment surgery, found alleviation of dysphoria and steady improvement in psychological functioning, with well-being similar to or better than same-age individuals from the general population [69].

Similar positive outcomes have been observed in other countries, including the United Kingdom and the USA. A survey-based study on 20,619 transgender adults in the USA found that those who received puberty suppression had lower odds of lifetime suicidal ideation compared to those who desired puberty suppression but did not receive it [70]. While the majority of adolescents with GD who start GnRHa subsequently initiate GAHT, GnRHa use is not associated with increased subsequent GAHT use [55]. Additionally, a small percentage of cases discontinue GnRHa therapy, mostly due to remission of GD, highlighting the therapeutic value of this option in facilitating informed decision-making for adolescents [71, 72]. This is particularly significant considering that GAHT, compared to GnRHa, leads to rapid and partly irreversible effects [73].

The primary findings from key literature studies concerning GnRHa treatment in GD, along with associated outcomes and adverse effects, are summarized in Table 3 [11, 56, 69–72, 74–86] and 4 [15, 87–99], respectively. In these tables, a nonsystematic overview of the most relevant original scientific papers, clinical trials, meta-analyses and reviews published in the last 15 years, in the English language, on a specific topic, were considered. Single case reports and letters were excluded. The following keywords (alone or in combination) were considered: gender dysphoria, GnRH analogs therapy, gender-affirming hormonal treatment, complications, adverse effects, benefits, outcome. The electronic databases PubMed, Scopus were used for this research Table 4.

**Table 3:** Literature data on GnRH analogues treatment in gender dysphoria and associated outcomes

**First author**	**Year**	**Mean age at GnRH treatment (years)**	**Number of individuals treated with GnRHa**	**Country**	**Type of treatments**	**Outcomes**
*De Vries *[68]	2011	13.1 AMAB, 14.1 AFAB	70	The Netherlands	GnRHa	Significant decrease in behavioral and emotional problems of the total problem scale, the internalizing and externalizing scale of both CBCL and YSR. Percentage of adolescents scoring in the clinical range significantly decreased on the CBCL total problem scale and the internalizing scale of the YSR. Depressive symptom scores on the BDI-II significantly decreased and global functioning ratings on the CGAS significantly increased. No significant change was observed in TPI (anger) or STAI (anxiety).
*De Vries *[69]	2014	13.6	55	The Netherlands	GnRHa → GAHT → surgery	Improvements in psychological functioning evaluated with UGDS, global functioning (CGAS), depression (BDI), anxiety (STAI), anger (TPI)
*Costa *[74]	2015	16.5	35	United Kingdom	GnRHa	Improvements in mental health evaluated with UGDS and CGAS, with significantly better psychosocial functioning after 12 months of GnRHa compared with when they had received only psychological support
*Staphorsius *[11]	2015	>12	20	Belgium/The Netherlands	GnRHa	No significant changes in terms of psychological functioning (CBCL), cognitive function (executive function task)
*Vrouenraets *[75]	2016	15.8	12	The Netherlands	GnRHa	Most adolescents found it difficult to define an appropriate age limit for starting puberty suppression and saw it as a dilemma Most adolescents stated that the lack of long-term data did not and would not stop them from wanting puberty suppression Some adolescents were positive about the role of the social context, but others raised doubts about it.
*Nahata *[76]	2017	15.3	9	USA	GnRHa	Transgender youth face significant barriers in accessing appropriate hormone therapy: only 8 received insurance coverage for GnRHa; of 16 individuals who had been denied insurance coverage for GnRHa, 4 began GAHT
*Lopez *[77]	2018	14	92	USA	GnRHa	Utilization of histrelin acetate implants increased dramatically. AMAB are more likely to receive implants and also more likely to receive implants at an older age. Treated transgender individuals are more likely to be White when compared to central precocious puberty
*Giovanardi *[78]	2019	37.4	10	Italy	GnRHa	Interview results: - treatment protocol considered an opportunity to prevent the body dysphoria and social phobia trans-people experience with puberty. - present the need to focus more on internal and psychological aspects of GD
*Kuper *[79]	2020	14.9	148	USA	GAHT	Reduced body dissatisfaction (assessed via BIS). Modest initial improvements in mental health (depression and anxiety).
*Turban* [70]	2020	18-36	89	USA	GnRHa	Treatment with pubertal suppression among those who wanted it associated with lower odds of lifetime suicidal ideation when compared with those who wanted pubertal suppression but did not receive it.
*Becker-Hebly *[80]	2020	15.5	75	Germany	GnRHa CSHT	Improvements in psychosocial health outcome, measured with CGAS, psychosocial functioning (YSR/ ASR)
*Cantu *[81]	2020	15	80	USA	GnRHa GAHT	No change in psychosocial functioning, evaluated with PHQ-9, GAD-7, no change in acute distress or suicidality idea
*Perl *[82]	2020	14	48	Israel	GnRHa GAHT	Possible increase in DBP in transgender male adolescents upon GHRH administration, and testosterone treatment may restore it, but further larger studies indicated
*Carmichael *[83]	2021	13.6	44	UK	GnRHa	No change in psychological functioning (CBCL, YSR), Self-harm, BIS, HRQoL
*Van der Loos *[84]	2021	11-17	322	The Netherlands	GnRHa GAHT	Development of hip bone geometry in transgender adolescents resembled that of the experienced gender if the GnRHa treatment was initiated during early puberty. Participants starting GnRHa during mid or late puberty continued within the curve of their gender assigned at birth.
*Schulmeister *[85]	2021	11.5	92	USA	GnRHa	TGD youth treated with GnRH have heigh velocity similar to that of prepubertal children, but TGD youth who start GnRHa later in puberty have an high velocity below the prepubertal range
*Nos *[58]	2022	15.4	434	USA	GnRH → GAHT	GnRHa use not associated with increased subsequent GAHT use, clinicians can offer the benefits of GnRHa treatment without concern for increasing rates of future GAH use
*Tordoff *[86]	2022	15.8	104	USA	GnRH → GAHT	Gender affirming medical interventions associated with lower odds of depression and suicidality over 12 months
*Van der Loos *[67]	2023	15	876	The Netherlands	GnRH → GAHT	Trajectories in diagnostic evaluation and medical treatment in children and adolescents referred for gender dysphoria are diverse. Initiating medical treatment and need for surgical procedures depends on not only personal characteristics but societal and legal factors as well

*AFAB *Assigned female at birth, *AMAB* Assigned male at birth, *BIS *Body Image Scale, *BDI *Beck Depression Inventory, *BMI *Body mass index, *BMAD *Bone Mineral Apparent Density, *BMI *Body mass index, *BMD *Bone Mineral Density, *CBCL *Child Behavior Checklist, *CGAS *Children's Global Assessment Scale, *DBP *Diastolic blood pressure, GAD-7 Generalized Anxiety Disorder-7, GnRH GnRH analogs, GAHT Gender-affirming hormone therapy, *HRQOL *Health-related quality of life, *PHQ-9 *Patient Health Questionnaire-9, *PS*I Temple Presence Inventory, *STAI *State trait anxiety inventory, *TGD *Transgender and gender diverse, *UGDS *Utrecht Gender Dysphoria Scale, *YSR *Youth Self-Report

**Table 4:** Literature data on GnRH analogues treatment in gender dysphoria and adverse effects

**First author**	**Year**	**Mean age at GnRH treatment (years)**	**Number of individuals treated with GnRHa**	**Country**	**Type of treatments**	**Outcomes**
*Khatchadourian *[87]	2014	14.7	27	Canada	GnRHa → GAHT	14/15 AFAB individuals transitioned to testosterone 5/11 AMAB individuals received estrogen treatment 1 AFAB individual developed sterile abscesses (switched from leuprolide acetate to triptorelin and well tolerated) 1 AFAB individual developed leg pains and headaches, resolved without treatment. 1 individual gained 19 kg within 9 months, although BMI was >85 percentile before initiation of GnRHa
*Klink *[88]	2015	15	34	The Netherlands	GnRH → GAHT → surgery	Decrease in lumbar area BMD z score (for natal sex)
*Schagen *[89]	2016	14	116	The Netherlands	GnRHa	No clear results concerning changes in height SD score and body composition that during GnRHa treatment
*Vlot *[90]	2017	13.5 AMAB, 15.1 AFAB	70	The Netherlands	GnRH → GAHT	Decrease of bone turnover markers in transgender adolescents treated with GnRHa with unchanged BMAD of femoral neck and lumbar spine, whereas BMAD Z-scores of predominantly the LS decreased especially in the young AMAB
*Klaver *[91]	2018	15	192	The Netherlands	GnRHa → GAHT	During treatment, waist-hip ratio and body composition changed toward the affirmed sex
*Joseph *[15]	2019	13	70	United Kingdom	GnRHa	No significant change in the absolute values of hip or spine BMD or lumbar spine BMAD after 1 year on GnRHa and a lower fall in BMD/BMAD Z-scores after the second year
*Stoffers *[92]	2019	16	64	The Netherlands	GnRHa → GAHT	Increased in acne, hematocrit, decreased HDL cholesterol, and decreased BMD z-scores
*Schagen *[89]	2020	14	121	The Netherlands	GnRHa → GAHT	BMAD z-scores decreased during GnRHa treatment and increased during gender-affirming hormone treatment
*Lee *[94]	2020	11.5	95	USA	GnRHa	Lower BMD than reference standards for sex designated at birth
*Klaver *[95]	2020	14.9	192	The Netherlands	GnRHa → GAHT	No increased cardiovascular risk upon GnRH treatment, evaluated with BMI, systolic blood pressure, diastolic blood pressure, glucose, homeostatic model assessment for insulin resistance, and lipid values
*Nokoff *[96]	2020	12	17	USA	GAHT	Transgender adolescents undergoing therapy show a body composition intermediate between BMI-matched cisgender males and females. Possible consequences for the cardiometabolic health
*Navabi *[97]	2021	15	198	USA	GnRHa	Lower BMD without evidence of fractures or changes in BMI z score

*AFAB *Assigned female at birth, *AMAB* Assigned male at birth, *BMI *Body mass index, *BMAD *Bone Mineral Apparent Density, *BMD *Bone mineral density, *GnRH *GnRH analogs, *GAHT* Gender-affirming hormone therapy, *TGD *Transgender and gender diverse, *SD *Standard deviation

#### Ethical positions and Italian rules

In April 2018, the AIFA sought guidance from the National Committee for Bioethics (NCB) regarding the ethical considerations surrounding the use of GnRHa in treating adolescents with GD. The NCB, while assessing the risks and benefits associated with off-label use, emphasized the paramount importance of addressing the significant distress experienced by adolescents with GD. This distress often manifests as elevated risks of suicide, self-harm, and heightened levels of depression and anxiety. Consequently, when psychological, psychotherapeutic, and neuropsychiatric interventions prove inadequate, the NCB deemed the use of GnRHa as justified to aid adolescents in navigating this intricate and challenging situation. The committee advocated for a cautious approach, recommending the judicious use of the drug in carefully selected cases, subject to individual evaluation [67]. Additionally, akin to the approach taken with other medications such as orphan drugs, the NCB recommended establishing policies to ensure equitable and uniform access to triptorelin at the national level by the National Health System (NHS), in order to ensure equal distribution, given the cost and the prolonged duration of therapy [67].

In line with these directives, the AIFA issued a resolution on February 25, 2019, incorporating depot triptorelin into the list of drugs covered entirely by the National Health Service, as per Law No. 648 of December 23, 1996, for use in specific cases of gender dysphoria (Resolution no. 21756/2019 of February 25, 2019) [53].

According to this resolution, the criteria for administering GnRHa in Italy encompass:

1. Confirmation of Tanner stage 2-3 puberty, verified by sex steroid levels indicative of appropriate pubertal progression.
2. Confirmation of gender dysphoria diagnosis per DSM-5 criteria by a multidisciplinary team comprising specialists in child and adolescent neuropsychiatry, pediatric endocrinology, developmental psychology, and bioethics.
3. Onset or exacerbation of symptoms coinciding with puberty onset.
4. Stabilization of any concurrent psychopathologies or medical conditions that could impede the diagnostic or therapeutic process of gender dysphoria.
5. Lack of efficacy of psychological, psychotherapeutic, or neuropsychiatric interventions.
6. Informed consent obtained from the adolescent and their parents or guardians.

In 2020, through two resolutions on September 23, the AIFA expanded the list to include testosterone, testosterone undecanoate, testosterone enanthate, testosterone esters, estradiol, estradiol hemihydrate, estradiol valerate, cyproterone acetate, spironolactone, leuprolide acetate, and triptorelin for use in the virilization process of transgender men (Resolution no. 104272/2020 of September 23, 2020) [98–100] and transgender women (Resolution No. 104273/2020 of September 23, 2020) [98–100], respectively.

According to these resolutions, the criteria for GAHT in Italy include:

1. Diagnosis of gender dysphoria/gender incongruence per DSM-5 [29] or ICD-11 [28] criteria, confirmed by a multidisciplinary specialist team.
2. Capacity to make an informed decision after receiving comprehensive information and providing consent to treatment.
3. For minors, consent to treatment from both parents or legal guardians in compliance with current regulations concerning minors (Article 3 of Law No. 219/2017).

Since 2015, surgical procedures altering primary sexual characteristics are no longer mandatory in Italy for legal sex rectification [10, 98]. Candidates for surgery should not exhibit significant medical or mental health concerns [52]. Before surgery, assessing the candidate's resilience is advisable to prevent decompensation in the event of complications or to assist individuals in managing post-surgery self-care. Therefore, a comprehensive informed consent process prior to surgery is recommended [52].

#### Pros and cons of GnRH analogues treatment for gender dysphoria

The decision regarding treatment must prioritize the child's best interests, following fully informed discussions with transgender and gender diverse youth and their families about the risks, benefits, and potential consequences of treatment.

Follow-up data examining the long-term risks or side effects of puberty blockers used to halt naturally occurring puberty in transgender youth, as opposed to precocious puberty, are still lacking. However, extrapolation from follow-up studies of blockers used for precocious puberty shows promising results. GnRHas have been utilized since 1981 for treating central precocious puberty, and their use is considered safe and effective, with no known severe long-term adverse effects. It should be remembered that GnRHas have been licensed for central precocious puberty based on relatively short open-label studies with small groups of patients because it was impossible and unethical to perform more robust studies (i.e. randomized controlled trials) [101].

The advantages and disadvantages associated with GnRH agonist treatment, as documented in the literature are outlined in Table 5 [8–17, 57, 97, 101–106].

**Table 5:** Pros and cons of GnRH agonist in gender dysphoria

**PROS**
• Alleviation of distress associated with puberty-induced physical changes, facilitating a calmer exploration of gender identity. • Prolongation of the assessment period and enhancement of diagnostic precision, allowing therapists to engage in ongoing psychological dialogue with adolescents, exploring all potential outcomes without the disruptive influence of pubertal changes. Moreover, this approach grants adolescents with gender dysphoria (GD) the opportunity to contemplate their gender identity and come to a decision regarding future gender-affirming interventions. • Prevention of irreversible physical changes that cause significant distress, obviating the need for potential future invasive medical and surgical procedures to align physical appearance with gender identity. • Reversibility of treatment: GnRH agonist (GnRHa) therapy does not constitute gender reassignment as it does not alter the body but maintains it in a neutral state. If GD resolves or the individual opts against gender transition, GnRH agonist therapy can be discontinued, allowing resumption of pubertal development aligned with biological sex. • Mitigation of adolescents seeking self-administration of hormones via alternative and hazardous methods (e.g., online medication purchases), thereby circumventing specialist supervision. • Attainment, upon reaching adulthood, of physical characteristics more congruent with the affirmed gender identity.
**CONS**
• The utilization of triptorelin may impact the cognitive maturation of adolescents. Experimental models suggest that sex steroids play a role in promoting cognitive maturity and are vital for normal brain development [8–10]. However, puberty suppression (PS) with GnRH agonists does not appear to have a detrimental effect on higher-order cognitive processes [11]. Moreover, the association between pre-treatment IQ and post-treatment educational achievement in transgender adolescents undergoing gender-affirming hormone therapy (GAHT), including GnRHa, seems comparable to that of the general population [12]. • The safety profile of puberty inhibitors is well-established in the context of early puberty. However, robust evidence regarding their safety in individuals with GD, including potential effects on fertility, remains lacking. Even if puberty is temporarily halted and subsequently allowed to progress, significant impacts on subsequent sexual function may arise, as the timing of hormone exposure during the peripubertal period is critical in determining adult sexual function [13]. • Psychological outcomes following GnRHa treatment may vary [103]. Moreover, there is limited knowledge about the use of GnRHa to halt normally timed puberty in youth with GD, as there are no long-term, longitudinal studies addressing this indication. • A potential reduction in bone mineral density among subjects treated with GnRHa cannot be dismissed [72]. Various studies have suggested that interrupting puberty may disrupt the anticipated trajectory of bone mass accumulation during adolescence. The extended clinical implications of failing to achieve typical bone mass accrual remain uncertain [14, 15]. • Possible short-term side effects of GnRHa treatment may include increased fat mass and body mass index, as well as reduced lean mass [16, 87] potentially impacting cardiometabolic health [105, 106].

### Criteria for centers referred to gender identity

A multidisciplinary expert team cognizant of the complexities of GD is mandatory in gender affirming care. As of today, access to dedicated clinical services and pathways for care and assistance is not uniform across the Italian national territory.

As reported by an Italian report [55], in 79% of centers TGD individuals receive the diagnosis of GD between 12 and 18 years of age and at least three healthcare professionals are involved (more often a child and adolescent neuropsychiatrist, a psychologist and a pediatrician). In only 3 centers a bioethicist is part of the team. Only 36% of centers include in the follow-up provided to TGD youths several investigations to evaluate the effect of the treatment and possible side effects, such as the evaluation of glucose metabolism, lipid profile, coagulation profile, bone mineralization and mental health assessment.

Considering the existing uncertainties surrounding the effectiveness and safety of hormonal treatment, it is deemed appropriate to identify a limited number of qualified centers across the national territory, not exceeding one/two per region, possessing the necessary skills and experience for the appropriate management of adolescents with GD. At present, the number of dedicated services is too low to meet the need of TGD child/adolescents.

According to international guidelines, accredited centers must meet specific criteria to ensure quality care for adolescents with GD. These centers must have:

- A skilled multidisciplinary team comprising child and adolescent neuropsychiatry, pediatric endocrinology, developmental psychology, bioethics.
- Endocrinologists experienced in prescribing GnRHa therapy and other hormonal treatments.
- Authorization to prescribe GnRHa therapy.
- Access to laboratory and radiological facilities for accurate diagnosis and follow-up.
- Transition care services from child/adolescent to adult services for persistent GD, including endocrinology and surgical expertise within the team.

In situations requiring it, a forensic medical opinion can specifically address legal matters such as discrimination, access to healthcare, and civil rights. The forensic interview and evaluation should take into account the prevalent perceptions held by the individual, as these perceptions can significantly influence their behavior and experiences across various life domains, including social, familial, educational, and occupational settings. These factors often play a critical role in an individual's mental health, resilience, capacity to function in daily life, and susceptibility to mental disorders. Furthermore, this evaluation should explore how GD could potentially affect the individual's ability to engage in legal proceedings, make informed decisions, and function effectively in society [107, 108].

Additionally, the proposal of a National Monitoring Commission, coordinated by the Italian National Institute of Health, is recommended, to which all diagnosed cases will be reported and included in a dedicated National Registry for individuals with GD [106]. This commission should:

- Formulate national guidelines and a specific care pathway for the diagnosis, treatment, and follow-up of adolescents with GD nationwide.
- Monitor the incidence of GD through a dedicated national registry.
- Monitor the effectiveness and safety of therapy.
- Evaluate and propose clinical trials to implement new therapeutic strategies.
- Conduct periodic audits of accredited centers.
- Conduct regular systematic reviews of evidence-based literature every two years.

The existing institutional website by the Italian National Institute of Health, called “Infotrans” [109], is dedicated to the well-being and health of transgender people and offers information on the available gender identity-related healthcare services. A specific section dedicated to qualified centers for adolescents should be implemented.

## Conclusions

In conclusion, pediatricians establish care with patients at birth manage individuals throughout childhood, adolescence, and young adulthood and are frequently the first formal contact for gender-nonconforming children and their families [108, 109]. Thus, pediatricians play a crucial role in understanding gender identity issues and providing appropriate care to individuals and families affected by GD [110, 111], while avoiding stigma and premature interventions. Receiving proper training is crucial for pediatricians to effectively address this challenge in collaboration with multidisciplinary teams of specialists [111].

The use of hormonal treatment in GD adolescents is a subject of ongoing debate, but it is essential to prioritize the health and well-being of TGD adolescents who are at higher risk of psychological challenges.

Treatment decisions should be made by healthcare professionals based on individual needs and circumstances, guided by scientific evidence rather than biases or ideologies.

Maintaining an evidence-based approach is essential to safeguard the well-being of TGD adolescents and to preserve the child's right to a promising future while recognizing that the individual child is the most impacted by decisions made.

Application of protection rights supports therapeutic interventions that protect the child from discrimination, provide the highest attainable standards of care, and deliver necessary access to it. However, the debate on respecting the rights of the child remains open [112].

Politicians and high court judges should address discrimination based on gender identity in legislation and support service development that aligns with the needs of young people.

Establishing accredited multidisciplinary centers capable of managing the diagnostic and therapeutic process for individuals with persistent GD is crucial to ensure quality care equitable and uniform access to hormonal treatment at the national level.

Furthermore, accredited centers can be beneficial for maintaining an ongoing dialogue with institutions regarding bioethical and medicolegal issues. Addressing these issues constructively and openly could improve the decision-making process while safeguarding the rights of children.

## Data Availability

Not applicable.

## Abbreviations

AFAB: Assigned female at birth
AIFA: Italian Medicines Agency
AMAB: Assigned male at birth
CGD: Childhood Gender Diversity
DEXA: Dual Energy X-rays Absorptiometry
DSM: Diagnostic and Statistical Manual of Mental Disorders
GAHT: Gender-affirming hormonal treatment
GD: Gender Dysphoria
GI: Gender Incongruence
GnRHa: GnRH analogs
IAP: Italian Academy of Pediatrics,
ISAM: Italian Society of Adolescent Medicine
ISCAN: Italian Society of Child and Adolescent Neuropsychiatry
ISP: Italian Society of Pediatrics
ISPED: Italian Society for Pediatric Endocrinology and Diabetes
PS: Pubertal suppression
TGD: Transgender and gender diverse
WPATH: World Professional Association for Transgender Health
